# Molecular, Spatial, and Field Epidemiology Suggesting TB Transmission in Community, Not Hospital, Gaborone, Botswana

**DOI:** 10.3201/eid2303.161183

**Published:** 2017-03

**Authors:** Diya Surie, Othusitse Fane, Alyssa Finlay, Matsiri Ogopotse, James L. Tobias, Eleanor S. Click, Chawangwa Modongo, Nicola M. Zetola, Patrick K. Moonan, John E. Oeltmann, for the Kopanyo Study Group

**Affiliations:** Centers for Disease Control and Prevention, Atlanta, Georgia, USA (D. Surie, A. Finlay, E.S. Click, P.K. Moonan, J.E. Oeltmann);; Botswana-UPenn Partnership, Gaborone, Botswana (O. Fane, M. Ogopotse, C. Modongo, N.M. Zetola);; Centers for Disease Control and Prevention, Gaborone (A. Finlay);; Northrop Grumman, Atlanta, (J.L. Tobias)

**Keywords:** Mycobacterium tuberculosis, bacteria, tuberculosis, TB, genotyping, mapping, GIS, geographic information system, transmission dynamics, outbreak, nosocomial transmission, cluster, community transmission, transmission, Africa, Gaborone, Botswana, tuberculosis and other mycobacteria, epidemiology

## Abstract

During 2012–2015, 10 of 24 patients infected with matching genotypes of *Mycobacterium tuberculosis* received care at the same hospital in Gaborone, Botswana. Nosocomial transmission was initially suspected, but we discovered plausible sites of community transmission for 20 (95%) of 21 interviewed patients. Active case-finding at these sites could halt ongoing transmission.

Tuberculosis (TB) results from rapid progression of a recently acquired *Mycobacterium tuberculosis* infection or from reactivation of a remote infection (*1*). It is critical that recent *M. tuberculosis* infections be identified because TB is more likely to develop in persons with recent infections (*2*). Furthermore, the finding of recently infected persons suggests ongoing transmission of TB, which can be interrupted by prompt identification and treatment of undiagnosed cases (*3*). However, finding undiagnosed cases remains a challenge (*4*). Name-based contact investigations have traditionally been used for this purpose, but such investigations are resource-intensive (*5*), making them less practical in countries to which TB is endemic. Genotyping of *M. tuberculosis* has emerged as a complementary method to detect ongoing transmission because persons who have the same TB genotype may be involved in the same chain of transmission (*6*). Although this assumption is relatively reliable in low-incidence countries, it is yet to be determined whether genotyping in TB-endemic settings can similarly detect ongoing transmission.

We investigated a TB cluster of 24 patients with matching *M. tuberculosis* genotypes in Gaborone, Botswana, a city with a high number of TB cases (*7*). Because almost half of these patients received care at the same hospital, nosocomial transmission was suspected. We conducted an investigation to determine if TB transmission occurred among these patients within the hospital and to identify possible alternate sites of ongoing transmission of this TB strain.

**The Study**

During August 2012–January 2015, all consenting persons with TB at 26 facilities in Gaborone provided sputum samples for culture as part of the Kopanyo study (*8*). *M. tuberculosis* isolates were genotyped by 24-locus mycobacterial interspersed repetitive units–variable number tandem repeats (*9**).*

We assessed nosocomial transmission by reviewing dates of hospital visits for overlap among the 24 TB cluster-associated patients. The hospital’s electronic billing system was used to obtain all previous dates of admission, discharge, and visits to the accidents and emergency department that had occurred for these patients since 2004.

We interviewed each patient, using an investigation form (Technical Appendix), to learn their primary residence; contacts; places of work and worship; and other frequented locations, including bars and combi (minibus) routes used in the 6 months before diagnosis. Primary residences of patients were mapped by using global positioning system coordinates (*8*). Ethical approval was obtained from the University of Pennsylvania, US Centers for Disease Control and Prevention, Botswana Ministry of Health, and University of Botswana. 

We looked for epidemiologic links that might suggest ongoing transmission. An epidemiologic link was defined for patients having at least 1 of the following associations: overlapping visits at the hospital, living within 1 km of another cluster-associated patient (spatial link), frequenting the same locations as another cluster-associated patient, and naming another cluster-associated patient as a contact.

During the study, ≈60% of *M. tuberculosis* isolates from reported TB patients in Gaborone were genotyped. The cluster discussed in this report includes 24 (2.3%) of 1,033 total genotyped cases from Gaborone.

All patients had pulmonary disease involvement (Table 1). Ten (42%) had received care at the hospital since 2004; most visits occurred after October 2013 (Figure 1). Except for visits by 2 patients, no patients’ visits overlapped at the hospital. Patients V and X overlapped in the hospital for 3 days, albeit in separate buildings. Patient V was admitted to the hospital with a known diagnosis of TB and had started TB therapy the day before admission. Patient X was in the hospital for a week but did not start TB therapy until 13 days after patient V was admitted. No members of this cluster were healthcare workers.

**Table 1:** Patient and disease characteristics in a tuberculosis cluster, Gaborone, Botswana, 2012–2015

Characteristic	No. (%)*
Patient	
Sex	
M	14 (58)
F	10 (42)
Age, median y (range)	31 (15–55)
Primary residence in Gaborone	20 (83)
Alcohol use	4 (17)
Tobacco use	3 (13)
Cough	23 (96)
History of visit to the hospital	10 (42)
Died	1 (4)
Disease-associated	
Pulmonary	24 (100)
Extrapulmonary involvement†	2 (8)
Positive sputum smear at diagnosis	12 (75)‡
HIV infection	16 (67)
CD4 cells/mL, median (range)§	310 (14–700)
Receiving antiretroviral therapy at time of tuberculosis diagnosis	9 (56)

*Data are no. (%) for 24 patients except as indicated. †In addition to pulmonary tuberculosis, 2 patients also had extrapulmonary involvement (pleural and abdominal tuberculosis). ‡Sixteen patients had a sputum smear tested at diagnosis. §CD4 counts were available for 11 of 16 patients with HIV infection.

**Figure 1:**
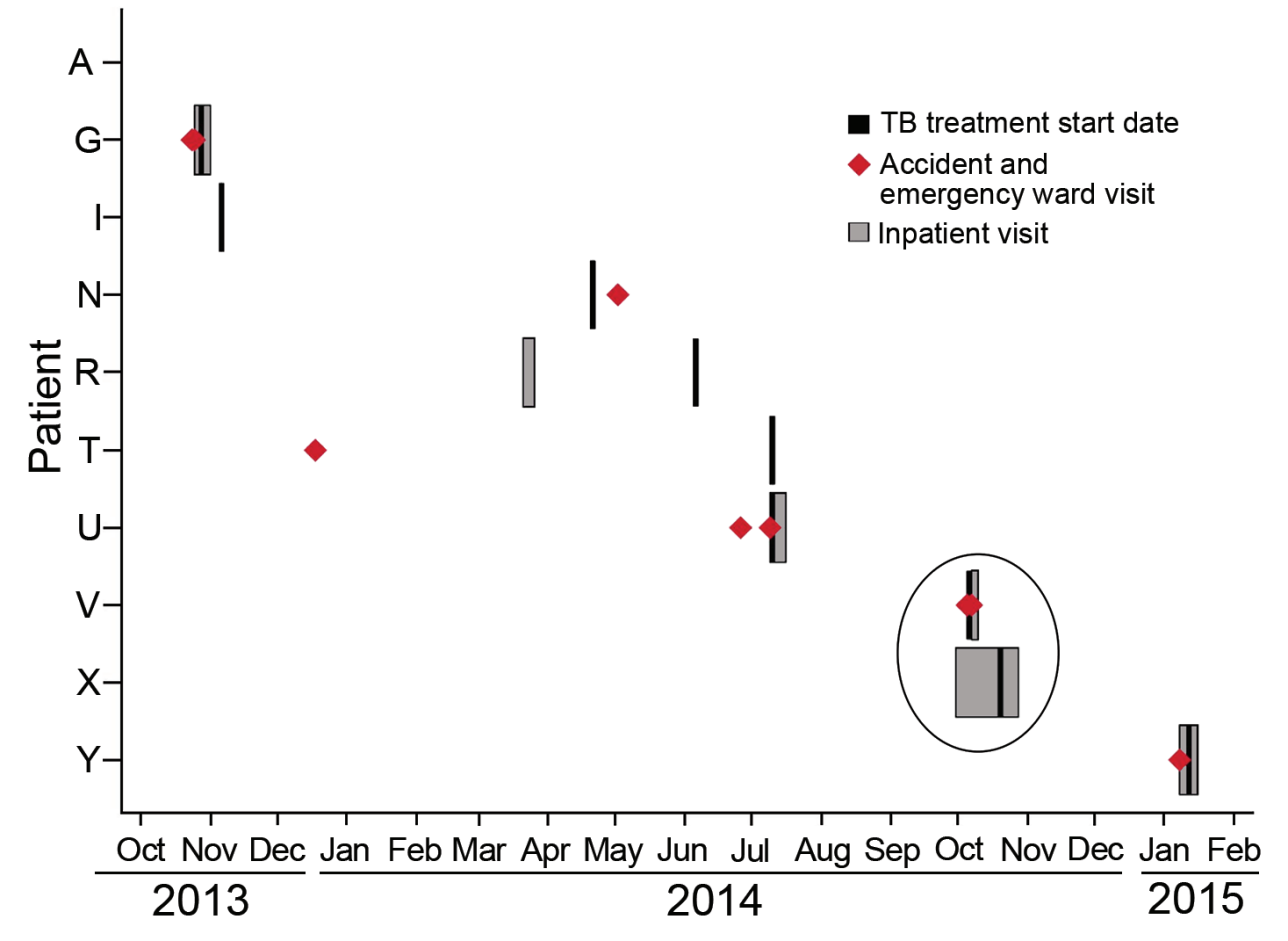
Timing of hospital visits and treatment for 10 tuberculosis (TB) cluster–associated patients, Gaborone, Botswana, 2013–2015. Patients were hospitalized or seen in the accident and emergency ward, and all had a history of such visits since 2004. Visits prior to October 2013 are not shown; these include visits in 2012 by patients A and I and additional visits by patients N, T, and U. None of the pre-October 2013 visits overlapped with those of other TB cluster–associated patients.

Twenty (83%) patients had a primary residence in Gaborone; 14 resided in 4 distinct neighborhoods (Figure 2, panel A). The spatial link definition (residing <1 km from another cluster-associated patient) was met by 13 (54%) patients. One patient (B) lived slightly >1 km from the nearest patient (Figure 2, panel B). Two of 4 spatially linked patients (C, D, I, and W) (Figure 2, panel B) did not name each other during enrollment when asked about contacts; when interviewed again as part of this investigation, these patients confirmed spending time together around the time of their diagnosis.

**Figure 2:**
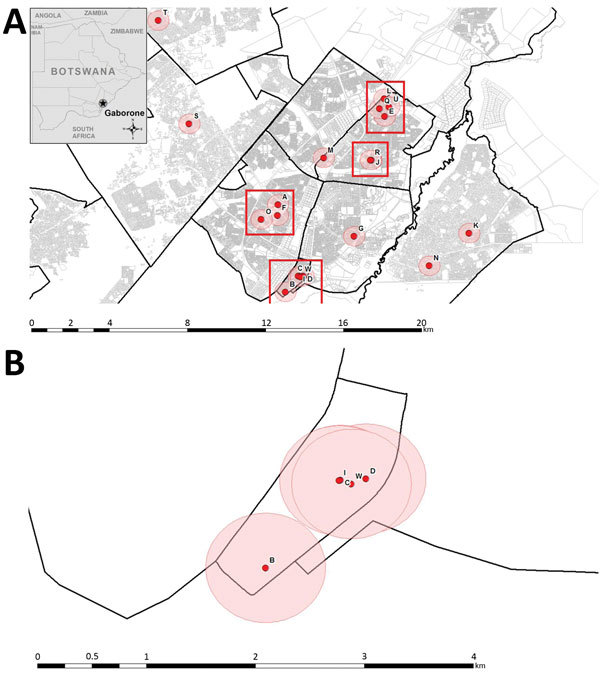
Residence-associated data for patients in a tuberculosis cluster, Gaborone, Botswana, 2012–2015. A) Primary residences of 20 patients are indicated by red dots. Inset map shows location of Gaborone in Botswana. Black lines demarcate neighborhoods; gray lines demarcate property parcels; pink circles represent 0.5-km radius around a patient’s residence; and red rectangles indicate presence of 14 patients in 4 distinct neighborhoods, 13 of whom had spatial links. Four patients who are not depicted on this map lived outside of Gaborone and did not have any spatial links between them. B) Primary residences of 5 patients who lived in the same neighborhood. Parcels locations were intentionally not shown to protect individual case anonymity. Geodata were sourced from Statistics Botswana (http://www.cso.gov.bw).

One patient died and 2 were lost to follow-up. Thus, 21 patients contributed to the remaining epidemiologic links obtained from interviews (Table 2). The median number of specific locations reported by patients was 6 (range 1–13). Of the 21 patients, 11 (52%) reported attending the same bar as another patient; 1 of these patients worked as a bartender. Site visits to 2 of the bars revealed an environment conducive to TB transmission (closed, poorly ventilated space crowded with patrons and employees). Similar crowded conditions were observed in combis, and 16 (76%) patients reported using the same combi route as another patient. Eight (38%) patients attended the same church as another patient, and 6 (29%) patients named each other as a contact, suggesting transmission could have occurred among them.

**Table 2:** Epidemiologic links between patients in a tuberculosis cluster, Gaborone, Botswana, 2012–2015

Link	No./no. total	%
Location		
Any	20/21*	95
Hospital A	2/24†	8
Combi routes	16/21	76
Spatial	13/24	54
Bars	11/21	52
Churches	8/21	38
Named contacts	6/21	29
No. links		
>2	14/21	67
>3	11/21	52
>4	3/21	14

*Only 21/24 cluster-associated patients were reachable for interview regarding epidemiologic links associated with contacts, combi routes used, and places of socialization and worship. †Hospital visits for 2 patients overlapped, but tuberculosis transmission between them probably did not occur because the patients were hospitalized in separate buildings and the time between tuberculosis exposure and treatment initiation (13 d) is an extremely short time for disease to develop.

## Conclusions

TB genotyping surveillance prompted a targeted investigation, which, when combined with spatial and field epidemiologic data, identified unsuspected locations of possible transmission. TB transmission from 1 cluster-associated patient to another at the hospital seemed unlikely. Although 2 patients’ hospital stays overlapped by 3 days, nosocomial transmission between them probably did not occur because the patients were hospitalized in separate buildings and the time from TB exposure and treatment initiation (13 days) is an extremely short time for disease to develop (*10*). Instead, the combination of links among patients suggests ongoing community transmission. Plausible sites of transmission included specific neighborhoods, bars, combi routes, and churches, transmission locations consistent with reports from other TB-endemic settings (*11**,**12*). These findings demonstrate the critical role that nonhousehold-based TB transmission plays in sub-Saharan Africa and highlight the need for identifying community-based TB transmission. A multidisciplinary approach (i.e., use of genotyping, spatial analyses, and interviews) provided us with locations where additional persons may be at risk for TB.

Our study has limitations. First, because samples from every TB case in Gaborone were not genotyped and interviews were not conducted with all persons in this cluster, key linkages between patients may have been missed. Second, underreporting of locations might have occurred due to patients’ inability to remember all locations visited; thus, some less frequented places where transmission might have occurred may have been missed. Third, we could not prove that patients who attended the same location interacted with each other at that location while infectious. Fourth, if a cluster-associated patient went to the hospital as a visitor, not a patient, their overlap with another cluster-associated patient could have been missed. Fifth, the extent to which the hospital infection-control program influenced our findings is unknown. Sixth, because 24-locus mycobacterial interspersed repetitive units–variable number tandem repeats characterizes only a portion of the *M. tuberculosis* genome, it is possible for 2 different strains to appear similar. Whole-genome sequencing could help confirm (or refute) the findings in this investigation.

Although genotyping is an imperfect tool for confirming transmission between patients, we know that numerous patients in this cluster visited the same community locations while they were potentially infectious, which alone could justify further active case-finding at these locations. With an estimated incidence of 385 TB cases/100,000 persons in Botswana (*7*), such clues are needed to focus TB control efforts. Active case-finding targeted at the most frequently visited community locations could help stop ongoing transmission of this strain.

Technical Appendix Investigation form used in a study of community-based tuberculosis transmission, Gaborone, Botswana, 2012–2015.
