# Supplementary material for: Molecular, Spatial, and Field Epidemiology Suggesting TB Transmission in Community, Not Hospital, Gaborone, Botswana
Source: Emerg Infect Dis. 2017 Mar;23(3):487–90. doi: 10.3201/eid2303.161183 (PMC5382725; doi:10.3201/eid2303.161183)
Supplement: Technical Appendix — Investigation form used in a study of community-based tuberculosis transmission, Gaborone, Botswana, 2012–2015. [file 16-1183-Techapp-s1.pdf]

Article DOI: http://dx.doi.org/10.3201/eid2303.161183

Molecular, Spatial, and Field Epidemiology Suggesting TB Transmission in Community, Not Hospital, Gaborone, Botswana

Technical Appendix

Kopanyo TB Cluster Investigation Form

Study ID: ☐☐☐☐☐	Interview Date: ☐☐/☐☐/☐☐☐☐ *dd mm yyyy*
	Site ID: ☐☐ *GA=Gaborone, GH=Ghanzi*
Interviewer's initials: ☐☐☐	MIRU Cluster ID: ☐☐☐☐

To be used at Cluster Investigation visit only

Instructions: Explain that you will be asking a series of questions to try to identify where we might be able to find other people who have TB. Acknowledge that the patient has already participated in an interview when he/she was first enrolled in the study. Some of the questions we are going to ask have already been answered. However, we may need to get additional details and will therefore repeat some questions. Reassure the patient that all answers will be kept confidential, and that the purpose of the interview is to learn information that can help stop the spread of TB and prevent other people from getting sick (emphasize protection of friends and family). List all responses on a separate sheet of paper. Thank the patient for his or her time and for speaking with us.

1. Name the places where the patient spent time in the six months before the diagnosis of this episode of TB

2. In the six months prior to diagnosis of this episode of TB, name the places where the patient has studied or worked

3. In the six months prior to diagnosis of this episode of TB, mention the places where the patient worships

4. In the six months prior to diagnosis of this episode of TB, mention the places where the patient goes for social activities, leisure (*e.g. bars/shebeens, shopping malls, homes other than primary residence).*

5. In the six months prior to diagnosis of this episode of TB, list all combi routes used by the patient

6. In the six months prior to diagnosis of this episode of TB, list the friends, family, church members, buddies with whom the patient spent significant time with

Comments
